# Disruption of the Ubiquitin-Proteasome System and Elevated Endoplasmic Reticulum Stress in Epilepsy

**DOI:** 10.3390/biomedicines10030647

**Published:** 2022-03-11

**Authors:** Sarah Poliquin, Jing-Qiong Kang

**Affiliations:** 1 Neuroscience Graduate Program, Vanderbilt University, Nashville, TN 37232, USA; 2 Department of Neurology, Vanderbilt University Medical Center, Nashville, TN 37232, USA; jingqiong.kang@vumc.org; 3 Department of Pharmacology, Vanderbilt University, Nashville, TN 37232, USA; 4 Vanderbilt Kennedy Center of Human Development, Vanderbilt Brain Institute, Vanderbilt University, Nashville, TN 37232, USA

**Keywords:** epilepsy, ubiquitin proteasome system, ER stress, proteostasis, chaperones

## Abstract

The epilepsies are a broad group of conditions characterized by repeated seizures, and together are one of the most common neurological disorders. Additionally, epilepsy is comorbid with many neurological disorders, including lysosomal storage diseases, syndromic intellectual disability, and autism spectrum disorder. Despite the prevalence, treatments are still unsatisfactory: approximately 30% of epileptic patients do not adequately respond to existing therapeutics, which primarily target ion channels. Therefore, new therapeutic approaches are needed. Disturbed proteostasis is an emerging mechanism in epilepsy, with profound effects on neuronal health and function. Proteostasis, the dynamic balance of protein synthesis and degradation, can be directly disrupted by epilepsy-associated mutations in various components of the ubiquitin-proteasome system (UPS), or impairments can be secondary to seizure activity or misfolded proteins. Endoplasmic reticulum (ER) stress can arise from failed proteostasis and result in neuronal death. In light of this, several treatment modalities that modify components of proteostasis have shown promise in the management of neurological disorders. These include chemical chaperones to assist proper folding of proteins, inhibitors of overly active protein degradation, and enhancers of endogenous proteolytic pathways, such as the UPS. This review summarizes recent work on the pathomechanisms of abnormal protein folding and degradation in epilepsy, as well as treatment developments targeting this area.

## 1. Introduction

Proteins are crucial to nearly every function in a cell, including both the general functions found in nearly every cell, such as energy production and DNA replication, as well as neuron-specific processes, such as maintenance of the membrane potential and synaptic vesicle release. Proteins must be folded properly in order to fulfill their functions, but protein folding is incredibly complex, and, thus, misfolding is common [1]. Up to one-third of newly synthesized proteins are degraded due to problems with synthesis or folding [1]. This inefficiency can pose problems, as not only is the native protein function lost by the misfolding, toxic protein aggregates may form [1]. Therefore, there are many quality control mechanisms in the cell to minimize these problems, ranging from methods of assisting folding, to machinery to degrade misfolded or aggregated proteins, or, in the case of severe proteostasis problems, programmed cell death.

A large proportion of newly synthesized proteins are folded in the endoplasmic reticulum (ER). The ER is equipped to facilitate proper folding, as its oxidizing environment promotes disulfide bond formation, and it contains many proteins that act as chaperones to further assist folding and act as quality control [1]. Complex oligosaccharides are added post-translationally to proteins, often further stabilizing them and enhancing interactions with chaperones [1,2].

Proteostasis is a delicate balance between protein synthesis and degradation, and disruptions of proteostasis are implicated in numerous diseases, including neurological conditions such as epilepsy. Epilepsy is a neurological disorder characterized by recurrent, unprovoked seizures and involves imbalanced inhibitory and excitatory neuronal circuitry. Neuronal function depends on a vast array of membrane proteins, such as voltage gated ion channels and neurotransmitter receptors and transporters, and membrane proteins in particular, are prone to rapid degradation, as their complex structure results in inefficient folding and assembly [2]. The GABA_A_ receptor (GABR) subunits are one example related to epilepsy, but this trend applies to other membrane proteins as well, such as cystic fibrosis transmembrane conductance regulator and peripheral myelin protein [2].

As mentioned above, GABR subunits are inefficiently trafficked to the cell surface, due to their complex structures, as well as the necessity of assembling with other subunits to form a complete pentameric receptor [2,3,4]. It is estimated that only 20–30% of newly synthesized GABR protein successfully reaches the cell surface [2]. Proper insertion of a full receptor into the membrane is further compromised by many epilepsy-linked mutations, such as α1(A322D) or γ2(Q390X), that result in ER retention of the misfolded protein or impaired heteropentamer assembly [4,5,6,7,8].

When a protein cannot be folded properly, it is degraded. Protein degradation is a crucial function in cells, to properly balance protein synthesis and to eliminate misfolded or mutated proteins [9,10]. There are two primary pathways in eukaryotic cells: macroautophagy and the ubiquitin–proteasome system (UPS). Macroautophagy, henceforth referred to simply as autophagy, involves the enveloping of the cargo in a double-membrane compartment, known as the autophagosome, which, therefore, can target large cellular components such as organelles, complex protein complexes, and protein aggregates [9,11]. In contrast, the UPS, which degrades the majority of intracellular proteins, primarily degrades individual proteins, as the target must be able to enter the narrow entrance of the proteasome [11,12]. These two pathways are interconnected, as they rely on shared components, such as ubiquitin, and disruptions to one pathway can alter the other [9,11].

## 2. The Ubiquitin-Proteasome System (UPS)

UPS mediated protein degradation is a constant and ongoing process that has a profound impact on cell survival and normal functioning. The UPS is critical in maintaining appropriate levels of regulatory proteins, such as those involved in the cell cycle, metabolism, and apoptosis, as well as in degrading misfolded proteins [9,12,13]. Dysfunction of the UPS is, therefore, associated with many diseases, ranging from cancer to neurological diseases, including epilepsy [9,12,14,15].

### 2.1. Unfolded Protein Response

In the case of misfolded proteins, the degradation process can begin in the ER with activation of the unfolded protein response (UPR). The UPR is activated in response to ER stress caused by factors such as accumulation of misfolded proteins or oxidative stress [16,17,18]. The function of the UPR is to restore proteostasis, which can be aided by several mechanisms: boosting the folding capacity of the ER by enlarging the ER and upregulating chaperones; decreasing the number of proteins to fold via increased ER-to-Golgi trafficking and halted protein translation; and/or enhancing protein degradation [1,16,19]. If these attempts fail, the UPR induces apoptosis [16,17,18,20]. There are three arms to the UPR—PERK, IRE1, and ATF6—but there is overlap in function, as all three can be both pro-apoptotic and protective [4,16,18]. The UPR plays a role in some of the neuronal damage observed in epilepsy: seizures can cause excessive ER stress and result in neuronal apoptosis [16].

### 2.2. Endoplasmic Reticulum Associated Degradation

One of the results of the UPR is enhanced protein degradation, through autophagy or through ER associated degradation (ERAD) [1,18]. ERAD is a major removal pathway for misfolded proteins, both wildtype and mutant, and is induced when an ER chaperone such as BiP recognizes a misfolded protein [1,5,21,22]. The misfolded protein is targeted for degradation via ubiquitination, which will be described in more detail below. After ubiquitination, the protein is retrotranslocated from the ER to the cytosol, where the proteasome is located [1,23]. The ATPase valosin-containing protein (VCP) provides the energy to move the protein through a retrotranslocation channel in the ER membrane [1,23]. Membrane proteins such as ion channels can be especially challenging to extract from the ER, as the hydrophobic membrane-spanning regions must be exposed to the cytosol during extraction [1]. ERAD plays an intrinsic role in epilepsies; for example, overactive ERAD and the subsequent loss of functional subunit proteins contributes to seizure pathology in some genetic epilepsies [2,22].

### 2.3. Proteasome

When proteins are targeted by ERAD, they are passed to the 26S proteasome. There are multiple types of proteasomes, but the 26S proteasome is found in all cell types and degrades damaged proteins [24]. The 26S proteasome is a large, complex cellular machine, consisting of at least 33 subunits and weighing 2.5 MDa [1,9,12,24]. It is composed of a 20S core particle and a 19S regulatory particle, and the core particle can be singly capped at one end by the regulatory 19S particle or doubly capped at both ends [1,9,10,24,25]. The heptameric 20S core is made of two outer rings of alpha subunits and two inner rings of beta subunits [9,10,24]. The beta subunit rings are the site of proteolytic activity, which takes three forms: caspase-like (β1), trypsin-like (β2), and chymotrypsin-like (β5) [1,9,10,24]. This results in polypeptides of 2–30 amino acids that are then further broken down by other processes [1]. The 19S regulatory particle is involved in substrate recognition, followed by unfolding of the substrate polypeptide and translocation into the catalytic 20S core [9,24]. The 19S particle is made up of a base of 10 proteins and a lid of nine more [9,10]. The expression of all these subunits is tightly regulated, and alterations to subunit expression has been observed in genetic epilepsy [9,26]. Excess excitatory transmission, such as occurs during a seizure or stroke, can also modulate the activity of the 26S proteasome [10].

### 2.4. Ubiquitin

**Figure 1:**
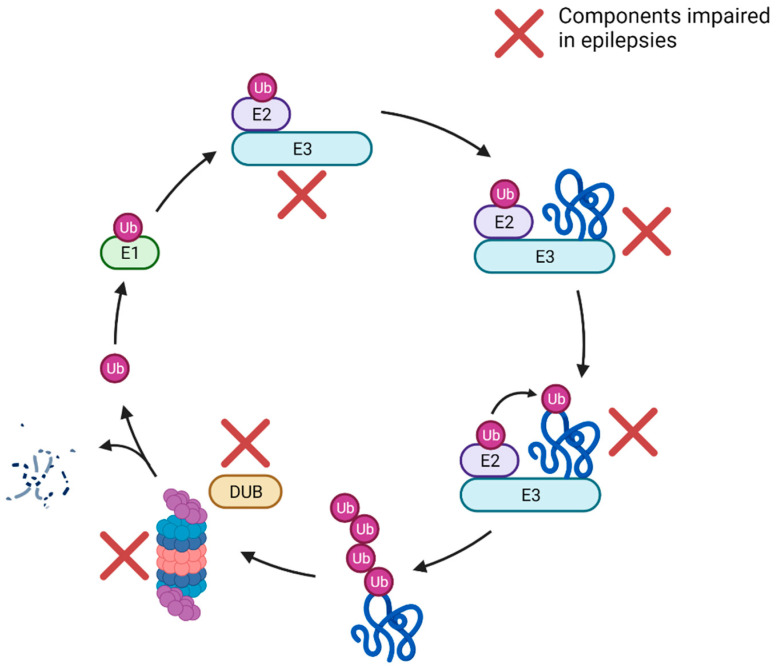
Epilepsy is associated with changes in the ubiquitination and proteasomal degradation of proteins. Under normal physiological conditions, a ubiquitin-activating E1 enzyme hydrolyzes ATP to bind to ubiquitin (Ub), and then transfers the ubiquitin to a ubiquitin-conjugating E2 enzyme. The substrate-specific E3 ubiquitin ligase recognizes a protein and transfers the ubiquitin from the E2 enzyme to the substrate protein. A polyubiquitin chain can then be formed by adding to the initial ubiquitin. The 26S proteasome recognizes the ubiquitinated substrate and hydrolyzes it into small peptides. A deubiquitinase (DUB) removes the ubiquitin molecules before they can be degraded, so that they can be reused. However, in pathological states, such as those that occur in epilepsy, several steps of this process can be compromised, including downregulation or mutation of E3 ligases, impaired substrate recognition or ubiquitination, inappropriate DUB activity, and dysregulation of the proteasome itself. These steps are indicated by a red X.

The first step in proteasome-mediated protein degradation is ubiquitination (Figure 1). The process of ubiquitination can be broken down into multiple stages: ubiquitin must first be activated by ATP with E1 enzymes, then transferred to ubiquitin-conjugating enzyme E2, and then moved to the target protein by E3 ubiquitin ligases [1,10,11,13]. The E3 ligases are substrate-specific, and over 600 have been identified in mammalian cells [1,11,27]. Some of these E3 ligases are directly implicated in epilepsy, and will be discussed in more detail below. Further ubiquitin proteins are added to lysine residues of the initial ubiquitin, resulting in a polyubiquitin chain [1,11,13,24].

Once ubiquitinated, the substrate is recognized by the proteasome, either through direct binding of the polyubiquitin chain to 19S subunits or though adaptor proteins that are associated with the proteasome [1,24]. In order to initiate degradation, there must also be an unstructured site on the ubiquitinated protein [24]. If there is no unstructured site, the stochastic unfolding and refolding can temporarily create an initiation site, but these proteins are more likely to escape the 19S subunits or 19S adaptors and, thus, not be degraded [24]. Unstructured proteins can directly enter uncapped 20S proteasomes, for proteolysis that is ubiquitin- and ATP-independent [9,24]. For ubiquitin-dependent degradation, the ubiquitin protein is removed from the substrate prior to degradation by deubiquitinases (DUBs), so that it can be reused [1,10,24]. DUBs can also remove the ubiquitin prior to association with the proteasome, allowing the substrate to avoid degradation [1,24].

## 3. The Role of the UPS in the Proteostasis of Synaptic Signaling

Efficient degradation of proteins is especially important for neurons, which, due to their post-mitotic state, have a more difficult time clearing toxic proteins [28]. When proteins are not degraded efficiently, prolonged ER stress and impaired UPS function can occur, and these factors are considered likely causes of neurodegeneration [10,16,29]. Proper UPS activity is crucial for correct neurotransmission: not only are both pre- and post-synaptic proteins regulated by the UPS, the growth of dendrites and spines, and the formation of new synapses, are also controlled by the UPS [13,14,25,27,30]. When excitatory and inhibitory neurotransmission is imbalanced, seizures can occur [31]. Thus, insight from the interconnection of synaptic signaling and the UPS may further understanding of epilepsy.

### 3.1. The UPS Is Affected by Synaptic Signaling

In order to ensure proper neurotransmission, there is complex feedback between the UPS and synaptic signaling: the UPS both regulates and is regulated by neuronal activity. Induced synaptic activity in cultured neurons results in increased ubiquitination in postsynaptic densities (PSDs), and elevated UPS activity was observed within minutes in dendrites [13,25]. Conversely, activity blockade via tetrodotoxin (TTX) results in decreased ubiquitination and UPS activity [13,25]. The decreased dendritic UPS activity after synaptic blockade is mediated by Ca^2+^ influx through NMDA receptors activating CaMKII, which phosphorylates the 19S subunit Rpt6 [25].

NMDA receptors, in particular, have been shown to have effects on the UPS. Bingol and Shuman (2006) showed that, in cultured hippocampal neurons, acute NMDA receptor activation resulted in a greater number of proteasomes in dendritic spines, primarily due to a six-fold decrease in the rate at which proteasomes exited the spines, but also aided by a subtler increase in the rate at which proteasomes were trafficked into the spines [32]. This change in localization is caused by active sequestering of proteasomes in the spines, mediated by an NMDA-dependent promotion of the association between proteasome subunits and the actin cytoskeleton [32]. In line with the findings above, the increase in proteasomes after excitatory stimulation was paired with augmented proteasomal activity, but there was a concomitant decrease in the amount of ubiquitinated proteins in dendritic spines [32].

Interestingly, a decrease in ubiquitinated proteins, which was accompanied by depressed 26S proteasome activity, was found in a study by Tai et al. (2010), after inducing long-term synaptic plasticity in cultured hippocampal neurons via NMDA exposure [12]. The decrease in both UPS activity and in the amount of ubiquitinated proteins indicates that protein ubiquitination was reduced, and this is supported by the downregulation of E3 ligases such as UBE3A and HUWE1 [12]. Another study found that excitotoxic levels of glutamate stimulation of cultured hippocampal neurons temporarily dampened 26S proteasomal activity, due to disassembly into 20S and 19S particles [10]. However, at this high concentration of NMDA, and in contrast to the previous study, NMDA resulted in more polyubiquitinated proteins, possibly due to the observed reduction of DUB activity [10].

### 3.2. Regulation of Synaptic Signaling by the UPS

While the UPS is influenced by neuronal activity, the UPS can also exert control over synaptic signaling, which has clear applicability to diseases of dysregulated synaptic transmission, such as epilepsy. The turnover of several crucial synaptic proteins has been demonstrated to be regulated by the UPS, including PSD-95, Shank, GKAP, AKAP, SPAR, and GRIP1 [13,25]. Activity-dependent alterations in UPS activity result in widespread changes to the protein expression profile in postsynaptic densities, and inhibition of the UPS replicates the shifts in protein expression seen in inactive synapses [13]. In addition, pharmacological inhibition of the proteasome in cultured hippocampal neurons resulted in a larger pool of recycling synaptic vesicles (vesicles that both exocytosed and endocytosed in response to K+ stimulation) [33]. This effect is activity dependent, as coapplication of TTX and antagonists of AMPA and NMDA receptors with the proteasome inhibitor abolished the change to vesicle pool size, although the blockers without proteasome inhibition had no effect [33]. This has important ramifications for synaptic function, as long-term UPS impairment could result in sustained changes to the pool of synaptic vesicles [33].

Various parts of the UPS, besides the proteasome itself, can influence signaling. FBXO2 is a substrate-recognizing component of an E3 ligase complex known to regulate NMDARs, and *Fbxo2*^−/−^ mice were shown to have altered NMDAR expression, with elevated GluN1 and GluN2A [34]. These mice were also observed to have a large increase in the number of axo-dendritic synapses, which are normally rare, and it is likely that these shaft synapses are the location of the excess GluN1 and GluN2A [34]. This dramatic promotion of unusual excitatory synapses could lead to epileptogenic activity [34]. The GluN2D NMDAR subunit, meanwhile, interacts with the E3 ligase Nedd4, and overexpression of Nedd4 abates GluN1/GluN2D receptor-mediated currents [35]. Nedd4 also regulates endocytosis and degradation of another excitatory receptor, mGlu7 [36]. Additionally, the kainite receptor subunit GluK2 is regulated by parkin, an E3 ligase implicated in Parkinson’s disease, such that mutant parkin leads to excess GluK2 and enhanced excitotoxicity in brain slices [37]. In combination with the effect of NMDA receptor stimulation on UPS activity discussed in the previous section, glutamate receptors, thus, have an intricate relationship with the UPS.

Proper proteasome balance is also necessary for other ion channels. The calcium-activated potassium channels in the K_Ca_2 family are responsible for the medium afterhyperpolarizing potentials (mAHP) that play a role in the regulation of neuronal excitability [38]. In chronically epileptic rats, K_Ca_2.2 was transcriptionally downregulated; likewise, in hippocampal slices incubated with the GABA receptor blocker gabazine, K_Ca_2.2 was reduced [38]. K_Ca_2.2 is a substrate of the E3 ligase UBE3A, so the decreased protein level was hypothesized to be due to UPS proteolysis [38]. Indeed, the reduction was blocked by MG132, and MG132 also lessened the epileptiform discharges in slices [38].

Another E3 ligase that regulates components of neurotransmission is SCRAPPER (encoded by *FBXL20*) [39,40,41]. SCRAPPER is enriched in the presynaptic membrane throughout the brain and controls levels of Rab3-interacting molecule 1 (RIM1) [39,41]. RIM1 assists in priming synaptic vesicles for release and is necessary for synaptic plasticity [39,41]. Unsurprisingly, then, as a result of upregulated RIM1 in *Scrapper*^−/−^ mice, there is a greater density of synaptic vesicles in the active zone [39]. Brain slices from *Scrapper*^−/−^ mice have reduced paired-pulse facilitation (PPF) and an increased mEPSC frequency [39]. Additionally, *Scrapper*^−/−^ mice have elevated glutamate and GABA concentrations throughout the brain [40]. Conversely, lentiviral overexpression of SCRAPPER in rats results in decreased RIM1 and is protective against seizures after pilocarpine administration [41]. SCRAPPER, therefore, plays an important role in regulating presynaptic neurotransmission.

## 4. ER Stress and Epilepsy

The epilepsies are a broad group of neurological disorders characterized by repeated unprovoked seizures, and together are one of the most common neurological disorders [16,42]. Approximately 30% of epilepsy patients do not respond to existing anti-epilepsy drugs (AEDs), which primarily target various ion channels [16,20]. Therefore, approaches that leverage other pathways have great potential for currently intractable seizures. As noted above, excessive excitatory input can alter the UPS, which in turn shifts protein expression [10]. Beyond immediate changes to synapse composition, impaired proteostasis can result in ER stress [16]. ER stress is an adaptive response to oxidative stress, ion balance disruptions, or misfolded proteins [1,16,18]. Genetic epilepsies, too, can directly cause ER stress, by the continued presence of misfolded protein [43,44]. Excessive ER stress can result in apoptosis, and indeed neuronal death is observed after seizures [16,18,45]. Managing ER stress, then, may alleviate some of the pathology associated with epilepsy.

### 4.1. Acquired Epilepsy

ER stress is seen in multiple animal models of epilepsy, as well as human brain tissue. In rats treated with kainic acid (KA) to induce seizures, multiple signs of ER stress were observed [45]. After KA administration, the ER chaperone BiP/Grp78 had enhanced expression, as did the ER stress-mediated proapoptotic factor CHOP [45]. A target of CHOP, TRIB3, became elevated at 24 h and remained high for days, as did other downstream apoptotic factors Bax and cleaved caspase 3 [45]. Similarly, in mice treated with the GABR inhibitor pentylenetetrazole (PTZ), to induce seizures, BiP and CHOP were elevated [20]. In addition, in hippocampal tissue resected from patients undergoing surgery for temporal lobe epilepsy, elevated levels of markers of ER stress were found [42]. Immunoreactivity for the KDEL motif shared by the chaperones BiP and Grp94 was stronger and more prevalent than in control brains, as was the immunoreactivity for another ER chaperone, calnexin [42].

This induction of ER stress is detrimental, as suggested by the electron micrographs of rat hippocampal tissue after KA administration, revealing swollen mitochondria and swollen rough ER, which is indicative of neuronal injury [45]. This was supported by greater TUNEL staining compared to control animals [45]. In the human tissue, various proapoptotic caspases, such as cleaved caspases 6, 7, and 9 were nearly absent in the control brains, but clearly expressed in brains with temporal lobe epilepsy [42].

### 4.2. Genetic Epilepsy

Genetic epilepsy can be directly linked to ER stress, independent of seizures. A Dravet syndrome-associated mutation, *GABRG2(Q390X)*, results in intracellular accumulation of misfolded protein [43,44]. This triggers ER stress, as shown by heightened expression of the ER-stress-induced pro-apoptotic factor GADD153/CHOP [43,44]. In transfected cells, the induction of GADD153 is correlated with increasing amounts of cDNA, suggesting that the accumulation of this overly-stable misfolded protein has negative consequences [44]. Indeed, neuronal death is seen in *Gabrg2^+/Q390X^* mice [43]. Elevated cleaved caspase 3, a marker of cell death, is detected in mice as early as 5–6 months, and more prominently at 12 months [43]. Additionally, the cleaved caspase 3 is colocalized with γ2 aggregates [43]. Increased TUNEL staining at 12 months also supports the idea of neurodegeneration, and, in 14–18-month-old *Gabrg2^+/Q390X^* mice, a decrease in the number of cells positive for NeuN (a neuronal marker) is observed in the somatosensory cortex [43]. Finally, the neurotoxicity is at least partially intrinsic to the direct effects of the misfolded protein, and not only a result of a lifetime of severe seizures, as cultured neurons from P0 pups also display higher amounts of cleaved caspase 3 [43]. Furthermore, other *GABRG2* truncation mutations, W429X and W461X, have been shown to mimic the effect of Q390X on ER stress, as expression of these mutant γ2 resulted in more GADD153, relative to wildtype γ2, and the induction of GADD153 corresponded to the amount of cDNA transfected into the cells [43,44].

Additional evidence for a connection between genetic epilepsy and ER stress comes from mutations in RNF13 [17]. RNF13 is a key activator of the JNK-mediated pathway of ER stress, via activation of the ER stress sensor IRE1α [17]. Three individuals with epilepsy and profound intellectual disability were found to harbor missense mutations in RNF13, and the study of patient-derived cells revealed increased ER stress signaling and ER stress-induced apoptosis [17]. Thus, these epilepsy-linked mutations produce a toxic gain-of-function in regard to ER stress [17].

## 5. Acquired Epilepsy and the UPS

The UPS is known to be impaired by the protein aggregates of neurodegenerative disorders, as well as by brain ischemia and traumatic brain injury [1,10,14]. Seizure disorders, too, are being shown to have a complex relationship with the UPS, which is not surprising, given how interrelated even normal neurotransmission and the UPS are, as discussed above (see Section 3) (Figure 1).

In two mouse models of induced status epilepticus (SE) (kainic acid and pilocarpine administration models), there was an accumulation of polyubiquitinated proteins in the hippocampus [14]. This was accompanied by diminished UPS activity, which was intriguingly most prominent in the regions most resistant to seizure-induced cell death (DG and CA1) [14]. Investigation into individual cell types showed that impaired UPS activity was first seen almost exclusively in neurons, but after 24 h, astrocytes became the affected population, with some contributions from microglia [14]. When mice had reached the chronic epilepsy stage, after convulsant administration, there was still an elevation in polyubiquitinated proteins, but proteasome activity was actually increased, and more 20S subunits were present [14]. At this later timepoint, neurons were again the cell type with dampened UPS activity, while astrocytes were not impaired [14]. Another study using a pilocarpine-treated rodent model also found time-dependent changes in the UPS [15]. In this model, ubiquitin was found to be downregulated in the acute (2 h post pilocarpine administration) and latent (3 weeks post administration) stages of epileptogenesis, but was upregulated in the chronic (8 weeks post administration) stage [15].

The E3 ligase Nedd4-2, which regulates ion channels, was also downregulated in both the acute and chronic stages, but not in the latent period in the pilocarpine-administered mouse model [15]. Pilocarpine also depresses another E3 ligase, SCRAPPER encoded by *FBXL20*, in mice [41]. Similarly, in hippocampal resection samples from patients with intractable epilepsy, there was decreased expression of SCRAPPER, but interestingly the synaptic protein it regulates, RIM1, was also reduced compared to control patients, underscoring a complex relationship between *FBXL20* and epilepsy [41]. E3 ligase downregulation has also been observed in vitro, after shifts in synaptic activity [12].

In line with the correlation of dampened UPS activity in areas with the least cell death, that study found proteasomal inhibition to be neuroprotective [14]. When cultured hippocampal neurons were exposed to an excitotoxic concentration of KA, proteasomal inhibition with small amounts of epoxomicin or MG132 was neuroprotective, and acute epoxomicin treatment was also shown to prevent neuronal death in two mouse models of epilepsy [14]. However, the other study found the opposite: when rats were pre-treated with MG132 before pilocarpine, they displayed heightened irritability and seizure frequency, and further loss of Nedd4-2 [15]. Additionally, neuronal cell loss in the hippocampus was aggravated by MG132 at all stages after SE [15]. The reorganization of mossy fibers of granule cells in the hippocampus appeared earlier and to a greater extent in the MG132 pretreated animals, compared to animals that only received pilocarpine [15].

A complementary study found that inhibition of a DUB exasperated neuronal injury after KA-evoked SE in mice [46]. Downregulation of the neuronally-expressed DUB ubiquitin carboxyl-terminal hydrolase isozyme L1 (UCHL1) was seen 24 h after SE, in line with findings that NMDA-mediated excitotoxicity downregulates DUBs in vitro [10,46]. Similar to the findings of regional differences above, UCHL1 was most depressed in the vulnerable CA3 region of the hippocampus [46]. Inhibition of UCHL1 with LDN-57444 downregulates monomeric ubiquitin and decreases UPS activity [46]; LDN-57444 treatment of mice prior to KA administration resulted in more neuronal death in the CA3 region of the hippocampus than in animals that did not receive LDN-57444, suggesting a neuroprotective role of UCHL1 [46]. The UPS, therefore, seems to have a complex relationship with seizures and neuron death.

## 6. Genetic Epilepsy and the UPS

**Figure 2:**
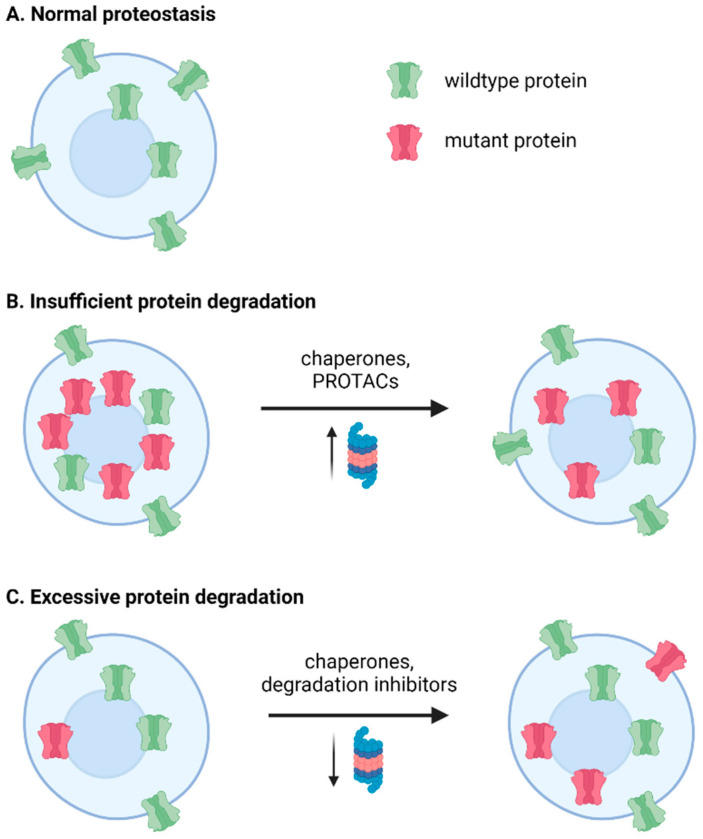
Rescue of dysregulated proteostasis in epilepsy. (**A**) Under healthy conditions, there is appropriate expression and trafficking of proteins. Here, a membrane channel is shown as an example. (**B**) Insufficient protein degradation can occur in epilepsies, due to factors such as aggregation-prone mutants, decreased E3 ubiquitin ligase activity, or loss of deubiquitinase function. Potential rescue mechanisms include chaperones, to prevent aggregation of mutant proteins, and PROTACs, to enhance degradation of specific substrates. (**C**) Excessive protein degradation is also seen in epilepsies, when a mutant protein with only partial loss of function is not permitted to reach its final location, in order to exert a function. Chaperones may help the protein fold properly, allowing proper trafficking, as would inhibition of the select components involved in the degradation of that protein.

Genetic epilepsy has an especially complex relationship to the UPS and proteostasis. In addition to the mechanisms discussed in the prior three sections—whereby elevated synaptic transmission can impact the UPS and therefore profoundly change the expression profile of synaptic proteins, and seizure activity can cause proapoptotic ER stress—the mutated proteins can exert their own effect on the UPS. Many mutations directly dysregulate components of the UPS, such as E3 ligases, while other epilepsy-causing mutations alter the proteostatic network with their rate of degradation (Figure 2).

### 6.1. Overactive ERAD in Genetic Epilepsy

Unnecessary degradation of mutant, yet still functional, protein is associated with several diseases, notably cystic fibrosis [1,4], but also several epilepsies connected to genes in the GABAergic pathway, such as *GABRA1*, *GABRG2*, and *SLC6A1* (Table 1). This overactive degradation results in lower levels of functional protein, potentially causing haploinsufficiency phenotypes, and also places a burden on the protein quality control mechanisms [1].

#### 6.1.1. GABRA1

The A322D mutation of the GABR subunit *GABRA1* causes autosomal dominant juvenile myoclonic epilepsy, and produces a mutant protein with partial loss of function [47]. When expressed in HEK293T cells with the partnering subunits β2 and γ2, peak GABA-evoked currents from α1(A322D)β2γ2 receptors were depressed to 10% of control cells containing wildtype α1 [48]. However, expression of α1(A322D) was also dramatically reduced, by 94% in HEK293T cells and 39% in transfected neurons [48,49]. What little protein remains is almost entirely retained within the ER [48]. Importantly, the misfolded α1(A322D) is still capable of interacting with β2 and γ2 subunits, and, therefore, traps the partnering subunits in the ER, resulting in a dominant-negative pathology [49]. This mutant subunit is subject to enhanced ERAD, as supported by a three-fold increase in association with the chaperone calnexin, compared to wildtype α1, and the decreased half-life [5,21]. Knockdown of various factors in the pathway of ERAD of α1 augmented levels of α1(A322D) and increased the GABA-evoked current [2]. Thus, the observed diminishment in ion current flow was only partially due to direct effects of the mutation on GABR function; some of the seizure pathology was due to trafficking problems and overactive ERAD [2].

#### 6.1.2. GABRG2

Overactive ERAD has also been documented for another GABR subunit, *GABRG2*. The R177G mutation linked with febrile seizures has impaired trafficking, having reduced surface trafficking and a lower proportion of mature glycosylation [22]. Additionally, intracellular γ2(R177G) was subject to higher rates of ERAD than wildtype [22]. However, the γ2(R177G) mutant protein is capable of assembling with α1 and β2 into pentameric receptors that insert into the plasma membrane, and these mutant receptors still have appreciable function (with the whole cell current density approximately 41% that of wildtype) [22]. Therefore, the accelerated ERAD decreases the pool of functional receptors and likely contributes to seizures [22].

Two mutations connected to generalized epilepsy with febrile seizures plus (GEFS+), γ2(R82Q) and γ2(P83S), are also retained with the ER [51]. When mutant subunits successfully are trafficked beyond the ER, however, they are capable of trafficking to the cell surface as functional receptors, albeit with poor efficiency [51]. This study did not investigate the degradation rate of these mutants, but the authors speculated that the mutant subunits are subject to enhanced ERAD, similarly to α1(A322D) and γ2(R177G) [51].

#### 6.1.3. GAT-1

Another gene in the GABAergic signaling pathway, *SLC6A1*, encoding GABA transporter 1 (GAT-1), is associated with ER retention of epilepsy-causing mutations. Several studies from the Kang lab found that the GAT-1 mutations manifesting with clinically diverse symptoms have similar cellular phenotypes [52,53,54]. Three mutations—G234S linked with Lennox-Gastaut syndrome, P361T associated with epilepsy and autism, and V125M identified in two siblings with epilepsy and ADHD—were found to have decreased total and surface protein expression, as well as diminished GABA uptake [52,53,54]. All three mutant proteins are partially retained in the ER, and the observed reduction in total protein expression is suggestive of enhanced ERAD [52,53,54]. Investigation of an additional 20 varied mutations also revealed trafficking abnormalities, as all 20 mutations had lower surface expression, while the total protein expression was not always reduced [55]. Further investigation into one mutation, S295L, associated with epilepsy and developmental delay, revealed prominent ER retention, which may result in ER stress [55].

### 6.2. E3 Ubiquitin Ligases and Seizures

A number of epilepsies and neurological disorders with comorbid seizures are due to genetic abnormalities that affect E3 ligases (Table 1). As E3 ligases are involved in almost all cellular process, E3 ligase dysfunction can result in a range of pathological changes, such as the accumulation of metabolic intermediates, decreased expression of proteasome subunits, and generally disrupted proteostasis of many key neuronal proteins (Figure 2).

#### 6.2.1. Lafora Disease

Lafora disease (LD) is a fatal, neurodegenerative disease belonging to the group of progressive myoclonic epilepsies [56,57,58,59,60]. It is generally caused by autosomal recessive mutations in *EPM2A* or *EPM2B*, although, recently, mutations in the related gene *PRMD8* have been implicated in LD [27,56,57,58]. *EPM2A* encodes the phosphatase laforin, and *EPM2B* (also called *NHLRC1*) encodes malin, an E3 ubiquitin ligase. Lafora disease is characterized by the presence of Lafora bodies, intracellular accumulations of insoluble glycogen-like carbohydrates, in the brain and other tissues [27,56,58,59]. Loss of function of malin or laforin diminishes the ability of the laforin–malin complex to regulate enzymes responsible for glycogen synthesis, resulting in Lafora bodies [27,56,59].

The exact mechanisms by which laforin or malin mutations result in epilepsy and neurodegeneration are unknown, but hypotheses include altered energy homeostasis and impairments of the UPS [27,57,58,59]. Due to the dysregulation of the glycogen synthesis machinery, improperly-branched glycogen-like polyglusans accumulate, and these are resistant to hydrolysis by α-amylase into glucose [27,59]. Glycogenolysis is the most important energy source for the brain, so reduced glycogenolysis can interfere with the basic processes of neuronal metabolism and result in hyperexcitable neurons [27,59].

Involvement of proteostasis pathways in LD is supported by the finding that Lafora bodies contain 6–28% protein, in addition to polyglusans [58,60]. The components of this protein in Lafora bodies include malin, ubiquitin, 20S proteasome subunits, and the Hsc70/Hsp70 chaperones [58]. Mutant malin expression can result in aggregates that recruit Hsp70 and are associated with cell death [58]. The sequestering of Hsp70 may be deleterious, as overexpression of Hsp70 abated malin aggregation and cell death [58]. Mutant malin also caused depressed UPS activity and a concomitant increase in ubiquitinated proteins [58]. Interestingly, in *Epmb*^−/−^ mice with no malin, elevations in ubiquinitated proteins is also seen, suggesting this phenotype is due to the loss of E3 ligase function, and not due to the presence of mutant protein [60].

#### 6.2.2. UBE3A Mutations

Deletion or loss-of-function mutations in maternally-imprinted Ubiquitin Protein Ligase E3A (*UBE3A*) result in the neurodevelopment disorder Angelman syndrome, while duplication of the maternal allele of the chromosomal region that spans the UBE3A gene, 15q11.2–q13.1, results in another neurodevelopmental disorder, Duplication 15q syndrome (Dup15q) [27,61]. Both Angelman syndrome and Dup15q often have comorbid seizures [27]. UBE3A is an especially interesting E3 ligase, as some evidence suggests it ubiquitinates the proteasome itself to regulate proteolytic activity [61]. At the cellular level, UBE3A disruptions are associated with many neuronal abnormalities, such as altered synapse formation and maintenance, dysregulation of ion channels, and disruption of the mTOR pathway [27,61]. The mechanism underlying epilepsy in Dup15q patients is not entirely clear, but some evidence points to a target of UBE3A, the Na+/K+ pump ATPα responsible for ion homeostasis [27]. For Angelman syndrome, seizure activity may be due to the loss of UBE3A in GABAergic neurons [27,61].

#### 6.2.3. CUL3-Associated Mutations

Cullin 3 (CUL3) is a scaffold for the E3 ligase class Cullin-RING ligases (CRL) [73,74]. Two patients with intellectual disability and infantile spasms were found to have missense mutations (V285A and R46Lfs*32) in CUL3, and analysis of the V285A mutant revealed a decreased ability to bind to KEAP1 (another component of the CRL complex), suggesting diminished stability of the E3 complex [73].

Mutations in another CUL3-associated protein, rho-related BTB domain-containing protein 2 (RHOBTB2), have been found in several patients with epileptic encephalopathy [74,75]. RHOBTB2 is both a substrate of CUL3 and an adaptor protein that assists CUL3 in targeting substrates for degradation [74]. The patient mutations showed higher expression of RHOBTB2 in heterologous cells, and overexpressing HA-CUL3 did not lower expression of the mutants like it did the wildtype protein, indicating a CUL3-mediated deficit in degradation [74,75]. Although the details of the relationship between excessive RHOBTB2 and seizures are not known, there may be dendritic abnormalities [75].

Additionally, a CUL3 substrate, potassium channel tetramerization domain-containing protein 7 (KCTD7), has been implicated in epilepsy [76]. Two siblings with PME and neuronal ceroid lipofuscinosis (NCL) have a homozygous R184C mutation [76]. This mutant protein has impaired trafficking, as decreased membrane staining and pronounced cytoplasmic aggregates were observed in heterologous cells, and has greatly decreased association with CUL3, which likely results in excess KCTD7, although the functional consequences of this are unknown [76].

#### 6.2.4. HECW2 Mutations

Multiple reports have identified mutations in the HECT, C2, and WW domain containing E3 ubiquitin protein ligase 2 gene (*HECW2*) in patients with severe epilepsy, intellectual disability, and hypotonia [77,78,79,80]. The encoded protein, HECW2, also called NEDD4-like ubiquitin protein ligase-2 (NEDL2), stabilizes p73, a tumor suppressor protein involved in neurodevelopment [77,78]. Although *Hecw2* knockout mice do not display CNS abnormalities, zebrafish with knockdown of the *HECW2* ortholog *hecw2a* had severe morphological abnormalities of the brain and spinal cord, as well as cell death [78]. Therefore, although the detailed mechanisms are unknown, HECW2 is another E3 ligase implicated in epilepsy pathogenesis.

#### 6.2.5. Fragile X Syndrome

Fragile X syndrome (FXS) is the most common inherited form of intellectual disability and is caused by loss of function of the *FMR1* gene that codes for the fragile X mental retardation protein (FMRP) [62]. In addition to autism and intellectual disability, some patients have epilepsy, and the *Fmr1* knockout mouse has neuronal hyperexcitability [27,62]. This hyperexcitability seems to be due to multiple factors. A study reported that, following PTX treatment of cultured neurons, *Fmr1*^−/−^ neurons failed to show the ubiquitination of the AMPA receptor subunits GluA1 and GluA2 that was seen in wildtype neurons [62]. This lack of homeostatic ubiquitination was mediated by impaired association of the E3 ligase Nedd4-2 with GluA1, as Nedd4-2 was inappropriately dephosphorylated after PTX treatment in the *Fmr1*^−/−^ neurons [62]. Nedd4-2 disruptions were also observed in pilocarpine-treated mice, supporting the role of Nedd4-2 in epilepsy [15]. Other studies in the *Fmr1*^−/−^ mouse have shown an absence of E3 ligase Mdm2-mediated ubiquitination of PSD95, leading to an excess of excitatory synapses [27].

### 6.3. Deubiquitinases and Seizures

Alterations to the ubiquitination process beyond the substrate recognition and ubiquitination steps mediated by E3 ligases can also result in seizures (Table 1). Deubiquitinases (DUBs) cleave ubiquitin moieties from proteosome substrates, so that the ubiquitin can be reused; shifts to DUB activity can, therefore, alter ubiquitin homeostasis (Figure 2), which has implications for seizures, as seen in the kainic acid mouse model of epilepsy discussed previously (see Section 5) [1,10,24,46].

#### 6.3.1. OTUD7A Mutations

15q13.3 microdeletion syndrome is characterized by a wide range of neuropsychiatric phenotypes, including epilepsy, developmental delay, and/or autism spectrum disorder [26,63]. The microdeletion spans two genes, *CHRNA7* (encoding the neuronal nicotinic acetylcholine receptor subunit alpha-7) and *OTUD7A* (ovarian tumor deubiquitinase 7A) [26,63]. Knockout of *Otud7a*, a DUB, in mice replicates much of the human phenotype and, thus, is believed to be the causative gene of 15q13.3 microdeletion syndrome [26].

Further support for the involvement of *OTUD7A* in epilepsy came when a patient with epileptic encephalopathy and severe developmental delay was found to have a homozygous missense mutation in *OTUD7A*, L233F [26]. Fibroblasts from this patient were cultured, and the proteasome activity in them was analyzed [26]. The patient-derived fibroblasts had greatly decreased 20S proteasome activity, which was found to be due to lower expression of the proteasome activator protein PA28-α and many 20S subunits [26]. The 19S regulatory subunits, however, were unaffected [26]. Additionally, the patient-derived fibroblasts showed an increase in polyubiquitinated proteins, indicating impairment in UPS function [26].

In 2021, another patient with epilepsy and developmental delay, as well as severe hypotonia, was reported to have a *OTUD7A* mutation, a homozygous de novo frameshift E375Dfs*11, in addition to a 15q13.3 microdeletion spanning part of *OTUD7A* [63]. To investigate the role of the frameshift mutation, a *Caenorhabditis elegans* model was created with a frameshift corresponding to the human mutation, in the *OTUD7A* homolog *otub-2* [63]. The homozygous *otub-2(ta112)* worms showed impaired locomotion, supporting the link between patient hypotonia and the E375Dfs*11 *OTUD7A* mutation [63].

#### 6.3.2. USP9X Mutations

Another deubiquitinase, Ubiquitin Specific Peptidase 9 X-Linked (USP9X), has been connected to genetic epilepsy. A patient with epileptic encephalopathy was found to have a de novo missense mutation (S1012P) at a conserved residue, and another patient with infantile spasms was found to have the missense mutations G1890E [81]. Interestingly, however, in the fruit fly *Drosophila*, loss-of-function mutations in the ortholog of *USP9X*, *faf*, were shown to confer resistance to seizures when crossed with the prickle model of epilepsy, *pk^sple^*/+ [81]. In the same vein, treatment of homozygous *pk^sple^* flies with the USP9X inhibitor Degrasyn/WP1130 was also seizure-protective [81]. The authors postulated that the discrepancy may be due to the *Drosophila* mutant alleles generating no protein product, while the human mutations do produce mutant protein, implicating a possible dominant-negative effect [81].

## 7. Protein Misfolding and Genetic Epilepsies

In addition to the seizure-related disorders with direct connections to the UPS discussed above, a number of other epilepsies have misfolded proteins that are prone to aggregation (Table 1). While alterations to proteasomal function may not yet have been found for all these diseases, given the strong link between neurodegenerative diseases, protein aggregates, and impaired UPS, it is likely that there are some similarities with the following disorders. A failure of proteostasis is, however, clear from the presence of aggregates.

### 7.1. STXBP1 Encephalopathy

Syntaxin binding protein 1 (STXBP1), or Munc18-1, is part of the machinery involved in the release of synaptic vesicles [64,65]. Mutations in this gene are linked with multiple neurological disorders, primarily developmental and epileptic encephalopathies [64,65]. Interestingly, some patients with STXBP1 encephalopathy, later develop juvenile-onset parkinsonism, and changes to STXBP1 levels are seen in Alzheimer’s disease, together suggesting a connection between STXBP1 and neurodegenerative diseases with protein aggregates [64,65]. Disease-causing missense mutations were predicted to be less stable than wildtype STXBP1 by in silico structural modeling, and this was corroborated by cycloheximide experiments in vitro [64,65]. Despite the decreased stability and shorter half-life of the mutant STXBP1, the mutants are prone to aggregation, as determined by Triton X-100 solubility and tryptic digestion assays [65].

Although the aggregates do not appear to be cytotoxic, they do represent a dominant-negative feature of mutant STXBP1: wildtype STXBP1 forms dimers, and the mutant proteins retain the ability to assemble with the wildtype protein [64,65]. Consequently, the aggregate-prone misfolded proteins also trap wildtype STXBP1 in aggregates, leading to lower expression of functional STXBP1 protein in transfected mouse neurons than expected for simple haploinsufficiency [64]. Since the mutant proteins are less stable, the association with wildtype protein also reduces the half-life of the wildtype STXBP1, further lowering the pool of functional protein [64]. Thus, improper folding leads to excessive protein degradation, as part of the pathomechanism for this severe epilepsy.

### 7.2. GABRG2 Mutations

Several mutations in *GABRG2* cause epilepsy, including *GABRG2(Q390X)* found in Dravet syndrome. The truncation of the γ2 protein results in a hydrophilic domain in place of the hydrophobic TM4, and this hydrophilic domain fails to stably insert into the membrane [43]. As a result, the mutation turns γ2(Q390X) from a membrane protein into a globular cytosolic protein [43]. Wildtype γ2 is already the GABR subunit most prone to self-dimerization, due to its proportionally high hydrophobicity, and the remaining exposed hydrophobic domains of γ2(Q390X) make this mutant especially prone to aggregation, due to interactions between the hydrophobic domains [43,66]. γ2(Q390X) is retained in the ER as stable dimers and larger oligomers, and has a dominant-negative effect on wildtype γ2, α1, and β2; trapping these partnering subunits in the ER, as well and reducing their surface expression [8,44,67]. The misfolded mutant is extensively ubiquitinated, more so than the wildtype, although total levels of all polyubiquitinated proteins are identical [44]. As previously discussed in the section on ER stress above, the γ2(Q390X) oligomers cause extensive ER stress and eventual neurodegeneration [43,44].

Although the clinical phenotype of Dravet syndrome connected to *GABRG2(Q390X)* is amongst the most severe epilepsies, other disease-associated mutations in GABR subunits share some of the molecular pathomechanisms. Several other truncation mutations in γ2 have been studied, and many form dimers and multimers, although not to the extent of γ2(Q390X). For instance, while γ2(R136X) is not prone to aggregation, W429X, which is linked to febrile seizures and generalized tonic-clonic seizures, does display moderate dimerization tendencies [44,66]. Yet, even without protein aggregation, R136X, W429X, and W461X have trafficking aberrations and are retained within the ER [6,44]. Non-truncating mutations, such as R82Q, also show this defect [7]. All of these mutations share with γ2(Q390X) the toxic ability to trap partnering α1 and β2 subunits within the ER, lowering surface expression and decreasing the relative amounts of mature, fully-glycosylated protein [6,44].

### 7.3. Familial Encephalopathy with Neuroserpin Inclusion Bodies (FENIB)

Familial encephalopathy with neuroserpin inclusion bodies (FENIB) is a neurodegenerative disorder with a range of symptom severity [23,68,69,71]. At its most severe, patients develop severe epilepsy in childhood [68]. FENIB is caused by mutations in neuroserpin, a primarily CNS-expressed serine protease inhibitor that is involved in synaptic plasticity [23,68,69,70,71]. The mutated proteins misfold and form neuroserpin inclusion bodies in the ER, and the most severe clinical phenotypes are associated with the mutants that polymerize most rapidly [23,68,71]. In part due to the correlation between clinical severity and presence of inclusion bodies, these inclusion bodies are believed to be part of the disease pathomechanism [23]. The mutant neuroserpin is targeted by ERAD for UPS-mediated degradation, but as the UPS becomes less efficient with age, the mutant protein can accumulate [23,68,70]. The E3 ligases implicated in mutant neuroserpin degradation are Hrd1, which also interacts with other epilepsy-causing proteins [3], and gp78 [23,68]. When a transgenic FENIB mouse model was crossed with a model genetic impairment of the UPS, twice as many neuroserpin inclusions were observed as in the control FENIB mouse, demonstrating the connection between the UPS and this protein-folding disease with seizures [70].

### 7.4. Progressive Myoclonus Epilepsy Type 1

Progressive myoclonus epilepsy type 1 (EPM1), also known as Unverricht-Lundborg disease, is a neurodegenerative disease caused by mutations in Stefin B (StB) [72,82]. StB, or cystatin B (CSTB), is a cysteine protease inhibitor [72,82]. While the majority of EPM1 cases have a repeat expansion in the promoter region of StB, which results in decreased transcription and, consequentially, lower protein levels, loss-of-function point mutations have also been found [72,82]. Two of these mutations, G4R and R68X, were investigated and found to form perinuclear aggregates that were associated with reduced cell viability in transfected cells [72]. The mutant aggregates costained, both with autophagy proteins and 20S and 26S proteasomes, indicating failure of the UPS to maintain proteostasis [72]. Interestingly, loss of function of StB (in a knockout mouse without mutant protein) resulted in the formation of aggregates of other proteins, and these aggregates contained almost no proteasome subunits and very few chaperones [82]. Therefore, lack of StB is associated with an impairment of the UPS [82].

### 7.5. KCNQ2 Epileptic Encephalopathy

*KCNQ2* encodes the K_V_7.2 subunit of the neuronal K_V_7 potassium channel, which is responsible for the M-current that suppresses neuronal excitability [31]. Mutations in the K_V_7 channel cause epilepsies of varying severity [31]. The K_V_7.2(M518V) mutation, identified in a patient with early-onset epileptic encephalopathy, was found to have poorer trafficking to the axon initial segment (AIS) and greater ubiquitination compared to wildtype K_V_7.2 protein [31]. Treatment with MG132 blocked the enhanced degradation of K_V_7.2(M518V) and resulted in aggregation of the mutant protein, demonstrating the involvement of the UPS in this disease [31]. Interestingly, co-expression with the partnering subunit K_V_7.3 also stabilized the mutant K_V_7.2 and enhanced aggregation [31]. The intracellular accumulation of K_V_7.2(M518V) caused cell shrinkage and nuclei condensation (features of apoptosis) in HEK293T cells [31]. In transfected neurons, K_V_7.2(M518V) also increased cell death [31]. These in vitro findings perhaps explain the MRI findings in a patient with the mutation, where there were small frontal lobes, a thin corpus callosum, and large ventricles, indicative of neuronal loss [31]. A lack of sufficient proteasome-mediated degradation of this mutant protein, therefore, may result in neurodegeneration as well as severe epilepsy.

### 7.6. Other Misfolded Proteins Associated with Epilepsy

*GRIN1*-related neurodevelopmental disorder is characterized predominantly by intellectual disability, epilepsy, and other neurological symptoms like hypotonia [83,84]. Missense mutations may have dominant-negative effects on NMDAR receptor assembly, as heterozygous null mutations such as deletions or truncations of *GRIN1* do not generally cause clinical phenotypes [83,84]. Instead, heterozygous missense mutations might suppress the pool of functional receptors by assembling with wildtype subunits in the ER and trapping them there [84].

*SPTAN1* encodes non-erythrocyte αII spectrin, and several patients with epileptic encephalopathies have been reported with *SPTAN1* mutations [85]. The spectrins form the cytoskeletal structure of the plasma membrane and are thus normally distributed evenly across a cell [85]. Imaging of fibroblasts from the patients showed instead aggregates of varying intensities, although aggregates were not observed in all patients [85]. Interestingly, however, despite the obvious presence of protein aggregates, the patient-derived fibroblasts did not seem to be more vulnerable to apoptosis, but neurons may have increased susceptibility [85].

## 8. Therapeutic Implications

### 8.1. Anti-Epilepsy Drugs That Impact UPS

A few existing, common AEDs have been found to have secondary effects on proteostasis and the UPS, in addition to their manipulation of neurotransmission (Table 2). Zonisamide is an AED that is also used in low doses as an add-on therapy for Parkinson’s disease [86,87]. Zonisamide helps to treat Parkinson’s disease by upregulating Sel1L, which in turn stabilizes the E3 ligase Hrd1, boosting the levels of Hrd1 [18,86,87]. This enhancement of Hrd1 is protective against cell death from tunicamycin or 6-OHDA, both of which induce ER stress [87]. Additionally, in type 2 diabetic mice, many ER stress-related proteins are elevated, and zonisamide treatment returned expression of most of the investigated proteins to control levels [18]. The cognitive impairment of the mice was rescued, potentially due to lessened ER stress-induced neurotoxicity [18]. Thus, in addition to the effects of zonisamide on ion channels and neuronal transmission, zonisamide may also be of use for epilepsy, due to this upregulation of Hrd1. Hrd1 is mechanistically linked to neurological disorders presenting with seizures: Hrd1, which is expressed in neurons in many regions of the brain, is involved in the degradation of epilepsy-causing mutant *GABRA1* and the mutant neuroserpin responsible for FENIB [2,3,23,68].

The AED valproic acid (VPA) is one of the most commonly used AEDs and has a complex mechanism of action [20]. In regard to proteostasis, VPA upregulates the transcription of the ER stress proteins BiP, Grp94, and calreticulin 26, and this yields greater protein expression [88]. VPA also prevented increases in BiP and CHOP in mice, after injection with the convulsant PTZ, as well as preventing apoptosis, as measured by TUNEL staining and cleaved caspase 3 expression [20]. VPA is also useful for abating ER stress in Wolfram syndrome, a multisystemic disease with several neurological components, caused by mutations of an ER membrane protein [89].

Carbamazepine (CBZ) is an AED and mood stabilizer that also has been shown to affect protein degradation. CBZ has been shown in vitro to reduce both mutant huntingtin and mutant α-synuclein, via autophagy enhancement [90]. The liver disease α1-antitrypsin deficiency (ATD) is a serinopathy, similarly to the neurodegenerative disease FENIB discussed in the previous section, and is likewise characterized by a deleterious accumulation of proteins in the ER; in this case, mutant α1-antitrypsin Z (ATZ) [69,91]. CBZ effectively enhances autophagy, to reduce ATZ aggregates and hepatic fibrosis [91]. In addition to autophagy, CBZ modulates proteasomal protein degradation: a study on the breast cancer protein Her-2 found that CBZ increases acetylation of Hsp90, which then lessens the ability of Hsp90 to interact with Her-2 [92]. This resulted in a decrease in Her-2 protein, which was blocked by MG132 treatment, showing that CBZ can promote protein degradation through multiple mechanisms [92].

### 8.2. Chaperones and Chaperone Upregulators

To relieve the burden on the UPS and restore proteostasis, chaperones that aid in protein folding may be of use in seizure-associated diseases, such as those discussed above (Table 2, Figure 2). Molecular chaperones are endogenous proteins, such as the various heat shock proteins that aid in protein folding, and can be induced by some small molecules, such as BIX (discussed below) [1]. Chemical or osmolyte chaperones provide a more favorable environment for protein folding, by altering the chemical environment of the ER, thereby stabilizing folded states and/or reducing aggregation tendencies [1,65]. Pharmacological chaperones such as 4-phenylbutyrate (PBA), meanwhile, directly interact with the misfolding-prone protein, instead of altering the general folding environment [1,65,94]. This may lower the free energy state of the protein to help it fold, or it may cover exposed hydrophobic regions of a misfolded protein to prevent aggregation [1,65].

#### 8.2.1. 4-Phenylbutyrate (PBA)

PBA is sold as the FDA approved drug Buphenyl for the treatment of urea cycle disorders, but has shown promise for a variety of neurological disorders with misfolded proteins [19]. PBA has several mechanisms of action. It is a hydrophobic pharmacological chaperone, preventing aggregation of misfolded proteins, by interacting with exposed hydrophobic regions [65]. PBA is also an inhibitor of histone deacetylases (HDAC), and perhaps via this effect, PBA upregulates the molecular chaperones BiP, Hsp70, and Hsp90 [1,60].

For aggregation-prone STXBP1 mutants, PBA not only increased the total levels of STXBP1, including wildtype, but it also decreased the fraction of detergent-insoluble protein, indicating that less of it was in aggregates and, thus, even more STXBP1 protein was functional [65]. Importantly, no negative effect was observed from overexpression of wildtype STXBP1, indicating that concerns from boosting the mutant protein, in non-aggregated forms, should be minimal [65]. Aggregates were also decreased in *C. elegans* expressing the various mutants, and localization of the protein within the ventral nerve cord was restored [65]. Furthermore, PBA rescued locomotion deficits in mutant worms [65].

PBA improved motor and neurobehavioral phenotypes in Lafora disease model *Epm2b*^−/−^ mice [60]. This was due to a reduction of polyglucosan inclusions and polyubiquitinated protein aggregates in the brain, which prevented neuronal loss and dampened reactive gliosis [60]. For ATP1A3 mutants associated with a range of neurological impairments, from hypotonia to severe infantile epilepsy, PBA promoted expression of the mutant protein in vitro by stabilizing them and promoting proper trafficking through the Golgi apparatus [19]. PBA also lessened trafficking defects for an ER-retained LGI1 mutant associated with autosomal dominant lateral temporal lobe epilepsy, LGI1(E383A) [101]. When the protein could be secreted, it was able to correctly bind to its receptor, and thus, PBA ameliorated the epilepsy phenotype in mouse models [101]. Creatine transporter deficiency syndrome has several neurological symptoms, including epilepsy and developmental delay, and is caused by mutations in the creatine transporter 1 (hCRT-1) [93]. These mutations result in a misfolded protein that is retained in the ER, and PBA treatment restored trafficking to the plasma membrane and creatine uptake functionality of several mutants [93]. In a non-epileptic neurological disorder, PBA also decreases ER stress resulting from dominant-negative mutations of Wolfram syndrome-causing WSF1 [89]. Additionally, PBA improves behavioral phenotypes in Parkinson’s disease and Alzheimer’s disease mouse models, and aids folding of the protein Pael-R implicated in autosomal recessive juvenile parkinsonism [86].

#### 8.2.2. Ambroxol

Ambroxol is a common mucolytic agent that crosses the blood–brain barrier and also acts as a pharmacological chaperone for acid β-glucosidase (GCase), the causative enzyme in Gaucher disease [94]. Gaucher is a lysosomal storage disorder that can have neurological presentations, including intractable epilepsy [94]. Ambroxol can bind to mutant GCase at neutral pH, such as found in the ER, to promote proper folding, but it dissociates at low pH [94]. As GCase localizes to the acidic lysosome, that means ambroxol does not interfere with function [94]. Ambroxol treatment has improved the symptoms of patients, both with neuropathic and non-neuropathic GD [94]. Of note, the efficacy of ambroxol for a given individual can potentially be screened using cultured fibroblasts from that patient [94]. For a patient whose fibroblasts had shown no benefit from ambroxol, ambroxol treatment was initiated regardless, due to the severity of his symptoms, but his symptoms did not improve [94]. Intriguingly, another patient who shared the same mutation (L444P) as the previous, ambroxol-resistant patient, did show improvement in fibroblasts [94]. This suggests that there are other factors at play, and underscores the usefulness of in vitro screening before initiating therapy in patients [94].

#### 8.2.3. Suberanilohydroxamic Acid (SAHA)

Suberanilohydroxamic acid (SAHA), also called Vorinostat, is an FDA-approved drug used for the treatment of cutaneous T-cell lymphoma [95]. Additionally, it has been extensively studied in vitro for GABR mutations linked to epilepsy, as it acts through BiP to stabilize misfolded proteins via upregulation of BiP transcription [50,95,96]. In HEK293T cells expressing α1(A322D)β2γ2 GABA_A_ receptors, SAHA application substantially upregulated α1, increasing protein levels 5-fold, and the half-life of a1(A322D) was tripled from 40 min to 125 min [50]. Partnering β2 and γ2 subunits were also upregulated [50]. Additionally, SAHA enhanced the trafficking efficiency of the α1(A322D) subunit, preventing degradation and, thereby, augmenting the amount of fully mature α1(A322D) [50]. Furthermore, the GABA-evoked currents were increased, showing that SAHA treatment resulted in functional α1(A322D)β2γ2 GABA_A_ receptors on the cell surface [50].

SAHA also rescues γ2 mutations that have reduced surface expression due to ER retention, such as N79S, R82Q, P83S, and R177G [95]. GABAergic currents for these mutations were partially or completely restored to wildtype currents, in regard to both amplitude and kinetics [95]. Interestingly, γ2(K328M), which does not have trafficking defects and only accelerates deactivation of the receptor, was not affected by SAHA treatment, further supporting the idea that SAHA functions as a chaperone for ER-retention-prone mutants [95]. SAHA, therefore, can directly ameliorate haploinsufficiency mechanisms, by boosting functional receptors, and prevent overburdening of the UPS by directing salvageable proteins away from ERAD. It is a promising option, due to already being FDA approved, non-cytotoxic, and able to cross the blood–brain barrier [50,95].

#### 8.2.4. Molecular Chaperone Bip Inducer X (BIX)

BiP inducer X (BIX), or 1-(3,4-dihydroxy-phenyl)-2-thiocyanate-ethanone, induces expression of the molecular chaperone BiP and prevents neuronal death from ER stress [4]. Cells exposed to the ER stress inducer, thapsigargin, did not show an increase in cleaved caspase 3 when also treated with BIX, and BIX halved the activity of caspases 3 and 7 [97]. BIX is also neuroprotective in vivo, increasing BiP and protecting mice from neuronal apoptosis after cerebral ischemia [97]. Additionally, the epilepsy-causing α1(A322D) GABR subunit displayed elevated surface expression after BIX treatment [4]. This was via enhanced trafficking efficiency of the mutant subunit, as indicated by an increase in the mature, endo H-resistant glycoform [4]. GABA-induced current was also increased, showing that BIX stabilization of the α1(A322D) subunit results in a greater number of functional receptors [4].

#### 8.2.5. DNP and DNEC

Dinoprost (DNP) is a synthetic analogue of prostaglandin F_2α_ that crosses the blood–brain barrier [3]. Dihydroergocristine (DHEC) is used to treat high blood pressure and dementia [3]. Both compounds were identified in a screening of a library of FDA-approved drugs as promoting total α1 subunit in HEK293T cells, and were subsequently tested on two epilepsy-associated mutations, α1(A322D) and α1(D219N) [3]. DNP and DHEC elevated surface expression of both mutants by 2–3-fold, compared to untreated cells, and this corresponded to greater peak GABA-induced currents [3]. The higher expression was due to decreased ERAD of the misfolded subunits, via suppressed transcription and expression of Hrd1 and Sel1L [3]. Additionally, the drugs enhanced the interaction of α1(A322D) with BiP and calnexin, resulting in a greater proportion of folded α1, which is then able to assemble with β2 [3]. Two epilepsy-associated γ2 mutants were also tested, R82Q and R177G, and DNP and DHEC likewise enhanced the incorporation of those subunits into functional GABA_A_ receptors [3]. Of note, DNP and DHEC worked additively with SAHA, implying different but complementary mechanisms [3]. Finally, the drugs were demonstrated as useful for neuronopathic Gaucher disease as well [3]. The most common mutation identified in Gaucher disease, GCase(L444P), is subject to excessive ERAD, and both DNP and DHEC increased the amount of functional GCase protein in patient-derived fibroblasts [3].

#### 8.2.6. Other Chaperones

Multiple other chemical chaperones have shown promise for neurological disorders with epilepsy. A class of oxysterol derivatives were demonstrated to act as pharmacological chaperones for NPC1, the protein causing Niemann-Pick type C1, a lysosomal storage disorder with many neurological manifestations, including seizures [102]. The oxysterol derivatives enhance folding and function of mutant NPC1, attenuating the accumulation of cholesterol in fibroblasts derived from a patient [102]. Another group of drugs—the azole anti-fungals itraconazole, posaconazole, and ketoconazole—have also been shown to chaperone NPC1 [102]. These compounds boost the expression of NPC1, by decreasing the rate of degradation [102]. Other osmolytic chaperones include sorbitol, used in STXBP1 models [65], and trehalose, under investigation for both Lafora disease and STXBP1 [60,65].

### 8.3. Protein Degradation Inhibitors

#### 8.3.1. Eer1

Valosin-containing protein (VCP) is part of the retrotranslocation machinery that transports polyubiquitinated proteins from the ER to the cytosol for degradation via the UPS [3,96]. Eer1 is an inhibitor of VCP, and application of Eer1 prevented the excessive ERAD linked to the GABR mutation α1(A322D), without impacting partnering subunits β2 or γ2 [96]. Eer1 did so by facilitating proper trafficking of α1(A322D), which led to an increase in the mature, post-ER glycoform, heightened surface expression, and increased GABA-induced peak current [96]. In line with suppression of the over-aggressive ERAD, less ubiquitination of α1(A322D) was seen after Eer1 treatment and the mutant had a longer half-life [96]. However, because the protein degradation pathways are interconnected, suppression of ERAD may boost autophagy [1]. Interestingly, the chaperone SAHA worked additively, further stabilizing and elevating functional α1(A322D) levels [96].

#### 8.3.2. Hsp90 Inhibitors

The heat shock chaperone Hsp90β recruits the glutamate transporter GLT-1 (also referred to as EAAT2) to the 20S proteasome [98]. In tissue from humans with temporal lobe epilepsy, Hsp90β is upregulated in astrocytes, a change also seen in rodent models of epilepsy [98]. This increase may be pathological, as Hsp90β inhibition by 17AAG elevated GLT-1 levels and glutamate uptake in astrocytes, and pretreatment of mice with 17AAG reduced number of seizures following injection of the convulsant kainic acid [98]. Importantly, in mice that had already developed chronic seizures, 17AAG also lessened the number of seizures and increased the percentage of seizure-free days [98]. Additionally, in the *Tsc1^GFAP^* CKO model of tuberous sclerosis complex, 17AAG decreased seizure frequency, although drug administration began weeks before normal seizure onset [98]. HSP990 is another potent inhibitor of Hsp90 proteins, with greater blood–brain barrier permeability and less toxicity than 17AAG [99]. Similar to 17AAG, HSP990 also upregulates GLT-1 in astrocytes, and produces greater latency to seizures when given to mice prior to the convulsant PTZ [99]. Spontaneous seizures were also reduced by HSP990 in the kainic acid models of temporal lobe epilepsy in both mice and cynomolgus monkeys [99].

### 8.4. PROTACs

A technology recently receiving a lot of attention is proteolysis targeting chimerics (PROTACs). PROTACs enhance the endogenous interactions between E3 ligases and their substrates, by utilizing a heterobifunctional molecule that consists of an E3 ligase ligand and a ligand to bind the substrate [24,30,100]. The ubiquitination of substrates by E3 ligases is the rate-limiting step of ubiquitination [27]. By facilitating this interaction, the E3 ligase can then more efficiently ubiquitinate the target protein, so it can then be degraded by the UPS [30,100]. PROTACs are exciting because they can hopefully be used to target so-called undruggable proteins—proteins that do not have a clear function to modulate with small molecules—by boosting degradation of the protein. PROTACs are already being utilized in clinical trials, for cancer and autoimmune disorders, and ones for neurological disorders are being developed [100].

Several PROTACs targeting tau have been discovered, including TH006, which reduced tau levels in vitro, and in a mouse model of Alzheimer’s disease, lessened the neurotoxicity of Aβ [30]. The first few discovered used a small peptide sequence as the ligands, but small-molecule PROTACs were discovered in 2019 that degraded tau in human neurons [30]. For Huntington’s disease, there are also small-molecule PROTACs that reduced the amount of huntingtin in patient-derived fibroblasts [30]. These drugs were also effective for degrading the other polyglutamine-expansion proteins, such as ataxin 3 and 7, responsible for spinocerebellar ataxia types 3 and 7 [30]. Parkinson’s disease associated A53T α-synuclein is degraded in vitro by multiple small molecule PROTACs [30]. These promising studies are likely only the beginning, so far only around 10 E3 ligases have been used, out of the 600 in the human body [100]; therefore, it is hopeful that many more PROTACs will emerge as more E3 ligases are investigated, such as the many E3 ligases addressed in this review.

## 9. Conclusions

The interrelationship between neuronal transmission, epilepsy, protein quality control, and the ubiquitin proteasome system is complex and multidirectional. For example, excitatory synaptic activity, such as after NMDA receptor activation, can result in complex changes to proteasome localization and activity and to ubiquitination patterns, thereby modifying the entire profile of proteins found in postsynaptic densities [10,12,13,25,32]. Loss of function of several E3 ubiquitin ligases, meanwhile, impacts multiple glutamate receptors, which results in enhancements to epileptic activity and excitotoxicity [34,35,36,37]. Elimination of an E3 ligase also causes changes to the pool of synaptic vesicles, as does inhibition of the UPS [33,39]. Neuronal excitability can also be regulated by the UPS via degradation of ion channels such as K_Ca_2.2 [38].

Unsurprisingly, given the effects even normal neuronal activity has on the UPS, status epilepticus can cause dramatic changes to the UPS, including downregulation of UPS activity and deubiquitinase expression, which vary in magnitude across brain regions and cell types [14,46]. The UPS plays a complex role in the neuronal death that is often observed after SE, as decreased UPS activity is found in the most resilient neurons, yet pharmacological UPS inhibition results in increased neuronal loss after SE [14,15].

As in the UPS, ER stress is also interlinked with neuronal loss and epilepsy. Epilepsy can cause impairments to the protein folding machinery in the ER, resulting in ER stress that can progress to apoptosis. Multiple animal models have demonstrated that seizures can cause elevations in ER stress-related factors, such as BiP and CHOP, and human epileptic brain tissue also shows increases in both ER chaperones and proapoptotic caspases [20,42,45]. ER stress can also arrive independently from seizure activity and instead be the result of misfolded and mistrafficked proteins, as seen in genetic epilepsies such as Dravet syndrome [43,44]. ER stress can even be causative of epilepsy, as mutations in a component of the ER stress pathway result in epilepsy [17]. Regardless of the cause of ER stress, however, it is still associated with neuronal death [17,43].

Epileptic disorders can be due to misfolded proteins that are degraded too quickly, or to misfolded proteins that accumulate in aggregates. Accelerated degradation of misfolded proteins that still maintain at least partial function is seen in *GABRA1*, *GABRG2*, and *SLC6A1* mutations [5,22,47,48,49,51,52,53,54,55]. Dampening this overactive ERAD allows the protein to traffic beyond the ER, where it can be retained, and become a functional membrane receptor [2,3,96]. It is likely that the elevated ERAD demonstrated for some of these mutations may be accompanied by ER stress.

Dysregulated degradation can also arise from mutations that alter the function of E3 ligases or their partnering proteins, or even via alterations to a substrate protein that impairs the ability of its associated E3 ligase to degrade it, as is the case for the CUL3 substrates, RHOBTB2 and KCTD7 [74,75,76]. Both too little, and too much, E3 ligase activity can result in seizures, as seen in the UBE3A-implicated genetic disorders Dup15q and Angelman syndrome [27,61]. Conversely, shifts in synaptic signaling can modulate levels of E3 ligases, underscoring the interconnectedness of neurotransmission and proteostasis [12]. Mutations to DUBs, too, can disrupt the UPS and result in epilepsy [26,63,81].

Further connections between epilepsies and compromised proteostasis can be found in the variety of genetic epilepsies that have intracellular protein aggregates. Proteins such as STXBP1 and Stefin B are prone to aggregation, even in wildtype forms, and epilepsy-associated mutations exacerbate aggregation [64,65,72]. Unsurprisingly, given the well-studied relationship of protein aggregates with neurodegenerative disorders, such as Alzheimer’s disease and Parkinson’s disease, some of these aggregate-forming epilepsy mutants also result in neuronal death, such as γ2(Q390X) and K_V_7.2(M518V) [31,43,44].

In terms of treatment, then, multiple modulations of proteostasis show promise for these many epilepsies. For proteins with partial function that are subject to accelerated ERAD, selectively inhibiting their associated components of the degradation pathway may be beneficial, such as VCP inhibition via Eer1 for α1(A322D) [96]. Conversely, when there is insufficient degradation, as is the case for fragile X syndrome or FENIB, the interaction between substrate proteins and the appropriate E3 ligase can be boosted by PROTACs [23,30,62,68,70,100]. Existing antiepileptic drugs with effects on the UPS, such as valproic acid, can potentially be leveraged for conditions with elevated ER stress. Various chemical chaperones, such as PBA, and inducers of molecular chaperones, such as BIX, show great promise for almost any genetic epilepsy. Chaperones aid protein folding, and, thus, can potentially both prevent mutated proteins such as STXBP1 from aggregating and stabilize functional mutants such as GAT-1 that are prone to rapid degradation. In addition, because many membrane proteins, including neurotransmitter receptors, are inefficiently folded, chaperones may be able to elevate the wildtype protein in cases of haploinsufficiency. Additionally, chaperones can reduce ER stress, as has been documented after SE, and thereby help prevent neuronal death.

## 10. Further Applications

Impaired proteostasis is a common feature in many other diseases, both neurological and not. Neurodegenerative disorders, including amyotrophic lateral sclerosis, Huntington’s disease, Alzheimer’s disease, Parkinson’s disease, and spinocerebellar ataxias are known to involve the accumulation of misfolded proteins and impairments of the UPS [30,64]. Decreased proteasome activity is also seen in Down syndrome, as well as overactivation of the unfolded protein response [103]. The neurohypophyseal form of diabetes insipidus is caused by mutations in vasopressin, which result in ER retention and coaggregation with wildtype protein, and eventual cell death [104]. A similar pathology is seen in the liver disease α1-antitrypsin deficiency [91]. Other disorders involving altered proteostasis include cystic fibrosis, type 2 long QT syndrome, and retinitis pigmentosa [4]. Thus, the study of aberrant protein trafficking, ER stress, and altered proteasomal activity has great promise to bring treatments to a number of epilepsy syndromes and many other disorders with similar pathologies of impaired proteostasis.

## Figures and Tables

**Table 1:** Proteostatic impairments in genetic epilepsies and neurological disorders with comorbid seizures.

Disease	Gene Involved	Proteostatic Impairment	Accumulation of Misfolded Proteins?	Reference
autosomal dominant juvenile mycolic epilepsy	*GABRA1*	overactive ERAD	no	[2,5,21,47,48,49,50]
febrile seizures	*GABRG2*	overactive ERAD	no	[22]
generalized epilepsy with febrile seizures plus (GEFS+)	*GABRG2*	overactive ERAD	no	[51]
*SLC6A1*-associated disorders	*SLC6A1*	ER retention	possibly	[52,53,54,55]
Lafora disease	*EPM2A, EPM2B*	loss of E3 ligase activity	yes	[27,56,57,58,59,60]
Angelman syndrome	*UBE3A*	loss of E3 ligase activity	no	[27,61]
duplication 15q syndrome (Dup15q)	*UBE3A*	excess E3 ligase	no	[27,61]
fragile X syndrome	*FMR1*	decreased ubiquitination	no	[27,62]
15q13.3 microdeletion syndrome	*OTUD7A*	decreased 20S expression	yes	[26,63]
*STXBP1* encephalopathy	*STXBP1*	protein aggregation	yes	[64,65]
Dravet syndrome	*GABRG2*	protein aggregation	yes	[8,43,44,66,67]
familial encephalopathy with neuroserpin inclusion bodies (FENIB)	*SERPINI1*	protein aggregation	yes	[23,68,69,70,71]
progressive myoclonus epilepsy type 1 (EPM1)	*StB*	protein aggregation	yes	[72]

**Table 2:** Drugs with effects on proteostasis and with potential use for epilepsies.

Drug	Mechanism	Reference
*antiepileptic drugs*		
zonisamide	upregulates E3 ligase HRD1	[86,87]
valproic acid	upregulates ER chaperones	[20,88,89]
carbamezepine	enhances proteasomal and lysosomal degradation	[90,91,92]
*chaperones*		
4-phenylbutyrate (PBA)	hydrophobic chaperone, HDAC inhibitor	[19,60,65,86,89,93]
Ambroxol	pharmacological chaperone	[94]
suberanilohydroxamic acid (SAHA)	upregulates BiP	[50,95,96]
BiP inducer X (BIX)	upregulates BiP	[4,97]
Dinoprost (DNP)	suppression of Hrd1 and Sel1L, facilitated substrate association with BiP and calnexin	[3]
Dihydroergocristine (DHEC)	suppression of Hrd1 and Sel1L, facilitated substrate association with BiP and calnexin	[3]
*degradation inhibitors*		
Eer1	VCP inhibitor	[96]
17AAG	Hsp90 inhibitor	[98]
HSP990	Hsp90 inhibitor	[99]
*degradation enhancers*		
PROTACs	promote interactions between E3 ligases and substrates	[24,30,100]
